# Socioeconomic determinants of nutritional behaviors of households in Fars Province, Iran, 2018

**DOI:** 10.3389/fnut.2022.956293

**Published:** 2022-09-26

**Authors:** Zohre Foroozanfar, Mohsen Moghadami, Mohammad Ali Mohsenpour, Anahita Houshiarrad, Azam Farmani, Mohsen Ali Akbarpoor, Razieh Shenavar

**Affiliations:** ^1^HIV/AIDS Research Center, Institute of Health, Shiraz University of Medical Sciences, Shiraz, Iran; ^2^Health Policy Research Center, Institute of Health, Shiraz University of Medical Sciences, Shiraz, Iran; ^3^Department of Clinical Nutrition, School of Nutrition and Food Sciences, Shiraz University of Medical Sciences, Shiraz, Iran; ^4^Student Research Committee, Shiraz University of Medical Sciences, Shiraz, Iran; ^5^Nutrition Research Department, National Nutrition and Food Technology Research Institute, Shahid Beheshti University of Medical Sciences, Tehran, Iran; ^6^Department of Community Nutrition, Shiraz University of Medical Science, Shiraz, Iran

**Keywords:** nutrition behaviors, socio-economic status, level of education, Iran, households

## Abstract

**Introduction:**

Households' dietary habits are affected by their environment and socioeconomic status (SES). This study aims to investigate eating behaviors and determine the factors affecting nutritional status in households in Fars Province in 2018.

**Method:**

In this cross-sectional study, urban and rural households were selected using the multistage sampling method. A questionnaire was employed to interview the mother or householder to record the demographic, SES, and dietary habits of the family for major food items commonly used. A logistic regression model was used to analyze the data. The *p*-value less than 0.05 was considered significant.

**Results:**

In total, 6,429 households participated in the study. The majority of households use traditional flatbread, low-fat milk, and liquid/cooking oil. Frying was the most prevalent method of cooking. Parents' level of education and SES were associated with type of consumed bread, milk and dairy, methods of food preparation, adding salt at the table, eating out, and fast-food usage. Parents' higher level of education was significantly associated with salt storage in optimal conditions and not using salt before tasting the meal.

**Conclusion:**

Most of the households had healthy practices, especially in consumption of certain oils and methods of preparing their food as well as keeping salt in an optimal condition and using iodized salt. The most important unhealthy nutritional behavior was high consumption of fast food and outdoor food, especially in urban regions. Unhealthy dietary habits were more prevalent in households with low household and regional SES. Both households and regions with higher SES had better dietary habits.

## Introduction

Recently, changes in lifestyle have raised the prevalence of chronic diseases such as cardiovascular disease, hypertension, diabetes, and cancers (1). The most important lifestyle determinants of non-communicable diseases (NCDs) are reduced physical activity and poor eating habits (2).

A healthy dietary habit includes various approaches to the consumption of different food groups, food items, and the preparation of daily meals. Several recommendations are available in order to stick to a healthy dietary habit and, consequently, to prevent NCDs, such as choosing vegetable oils over animal-based oils, consuming fruits and vegetables on a daily basis, and using low-fat dairy products (3). Moreover, the food preparation method is also a major factor affecting health (4). According to the nutritional transitions, fast-food consumption has been increased alarmingly, with its health consequences emerging (3).

In addition, the dietary habits of individuals and households are affected by their environment and socioeconomic status (SES). The association between SES and poorer health has been recognized, and inequalities in nutrition have been associated with inequalities in health. Higher SES environment, education, and income are linked to changing dietary habits, but not always in a desirable way (3). Populations in low SES probably are at a higher risk of unhealthy conditions due to the lack of access to healthcare and poor nutrient intake. Thus, given public health policies, the assessment of eating habits in each population is essential for preventing diseases and nutrient deficiencies (5).

A few studies have been conducted in Iran to comprehensively assess nutritional behavior and its possible determinants for different geographical areas in rural and urban populations. Therefore, we aimed to assess the nutritional behavior of households in rural and urban populations of Fars Province, Iran.

## Materials and methods

A cross-sectional study was designed to investigate the nutritional behavior of urban and rural populations of Fars Province, Iran. The study protocol was approved by the Shiraz University of Medical Sciences (SUMS), Fars, Iran, under the registration code: IR.SUMS.REC.13940598.

### Sampling

Households residing in the urban and rural areas of Fars Province, covered by the Health Department of SUMS, were included in the study.

**Figure 1:**
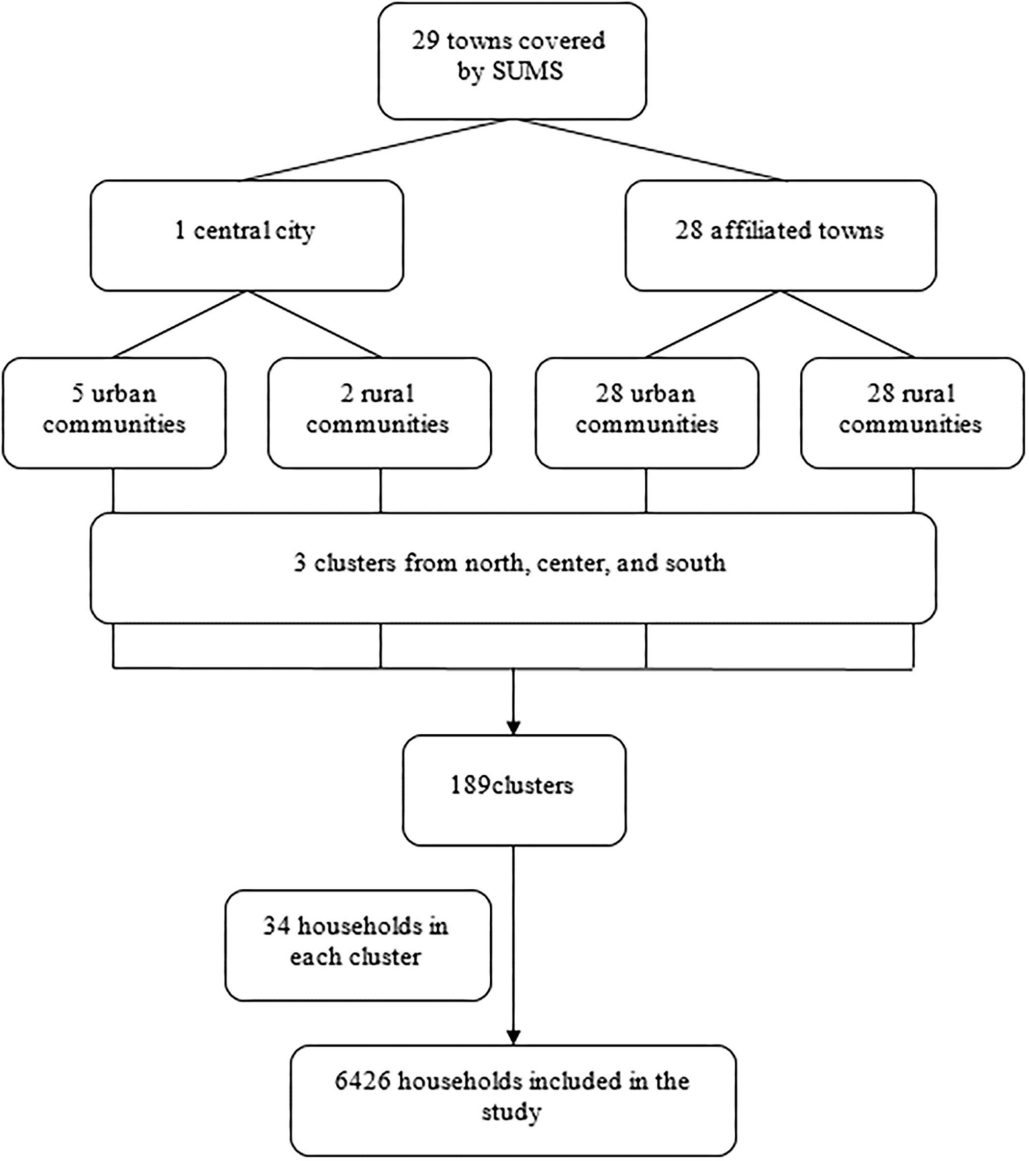
Sampling procedure of the study.

Fars Province, a populated province, is located in the southwest area of Iran. SUMS is one of the major universities of medical sciences in Iran, which is placed in Shiraz, the capital of Fars. SUMS Health Department covers 29 out of 36 towns in Fars Province (Figure 1). In this study, a multistage sampling method was used. In the first phase, all 29 towns were considered 29 stages (28 affiliated towns and Shiraz as the center). In the second phase, the towns were divided into two urban and rural communities. In addition, Shiraz was divided into 7 areas, including 5 urban communities (3 districts of Shiraz—northern, central, and southern, and 2 northern and southern suburbs of Shiraz) and 2 rural communities. Subsequently, by means of the maps, urban and rural areas were separated into three clusters of north, center, and south with a maximum spatial accuracy. Finally, we obtained 168 clusters from affiliated towns and 21 clusters from Shiraz. Then, 34 households in each cluster were included using a systematic random sampling method. In this stage, the first household was selected by random selection and sampling was continued from the right side of the house until reaching the predetermined sample size. Thus, a total of 6,426 households were included in this study. The mother of each household was considered the representative in order to respond to the interviewer, or, in the absence of the mother, the householder (the person in the household who is responsible for making decisions and earning money) was interviewed. Figure 1 depicts the sampling procedure of the study.

### Household assessment

A questionnaire was compiled to assess general and sociodemographic characteristics of the households. The questionnaire asked about the persons who were present in the household for the last 7 days continuously, head of the household, and his/her spouse's level of education (illiterate, elementary level, high school, diploma, graduate, and postgraduate), and job status (unemployed, farmer, full-time worker, daily worker, employed, self-employed, and retired for father and housewife, employed, and self-employed for mother).

The sociodemographic status of the household was calculated by considering the possession of 9 specific items, including home, personal vehicle, washing machine, LCD TV, dishwasher, refrigerator, handmade rug, laptop, and microwave. Based on the number of items possessed by households, the SES was categorized into three groups, namely, low (3 items or less), moderate (4–6 items), and high (more than 7 items) (6).

### Dietary assessment

A nutritional behavior questionnaire was used to investigate the overall food consumption habits of households (1, 7). Interviewers asked about types of consumed milk and dairy (low fat vs. high fat), bread (traditional flatbreads or homemade breads), and oil (different types of vegetable oils or animal oils), in addition to their consumed amount. The frequency of the consumption of vegetables and salad (weekly) and salt consumption habits were assessed by the questionnaire. Interviewers asked about iodized salt consumption, and in case of a positive response, its storage method within the household was investigated to indicate whether the storage is optimal or undesirable. In addition, the habit toward salt was recorded based on using salt at the table and adding salt to the meal before tasting it. Moreover, using dietary habit questionnaires, data were gathered about food preparation methods, including boiling, grilling, steaming, roasting, frying, or, other traditional methods, and consuming fast-food and packaged food.

### Statistical analysis

To describe the quantitative and qualitative characteristics (ordinal and nominal variables) of the households, descriptive statistical procedures including mean ± standard deviation (SD), frequency, and percentage were performed, respectively. To determine the contribution of food habits or consumption and place of residence, the chi-square test was used. In addition, the logistic regression model was used to estimate the association between sociodemographic characteristics and food habits or consumption. All statistical analyses were performed using the SPSS software version 22 (IBM, USA). A *p*-value of less than 0.05 was considered significant.

## Results

### Household general characteristics

A total of 6,429 households were included in the study, of which 5,826 (91.4%) were headed by a father. The mean household size was 3.91 ± 1.38, which was 4.01 ± 1.48 in rural areas and 3.80 ± 1.27 in urban areas. The characteristics of households, householders, and spouse's level of education and job based on place of residence are reported in Table 1.

**Table 1:** Socio-demographic characteristics of households by place of residence.

		**Total (*n* = 6,429)**	**Urban areas (*n* = 3,261)**	**Rural areas (*n* = 3,168)**
**Household size (mean** **±SD)**		3.91 ± 1.38	3.80 ± 1.27	4.01 ± 1.48
**Householder**	Father	5,826 (91.4)	2,975 (91.8)	2,826 (91.0)
	Mother	501 (7.9)	246 (7.6)	255 (8.0)
	Other	45 (0.7)	19 (0.6)	26 (0.8)
**Mother's level of education**	Illiterate	1,214 (19.0)	428 (13.2)	786 (24.9)
	Elementary	2,083 (32.6)	794 (24.5)	1,289 (40.9)
	High school	1,241 (19.4)	660 (20.4)	581 (18.4)
	Diploma	1,156 (18.1)	782 (24.1)	374 (11.9)
	Graduate	655 (10.2)	532 (16.4)	123 (3.9)
	Post graduate	44 (0.7)	43 (1.3)	1 (0.0)
**Father's level of education**	Illiterate	772 (12.8)	269 (8.8)	503 (16.9)
	Elementary	1,665 (27.5)	594 (19.3)	1,071 (35.9)
	High school	1,589 (26.3)	714 (23.3)	875 (29.4)
	Diploma	1,197 (19.8)	799 (26.0)	398 (13.4)
	Graduate	700 (11.6)	583 (19.0)	117 (3.9)
	Post graduate	127 (2.1)	111 (3.6)	16 (0.5)
**Father's job**	Unemployed	400 (6.6)	143 (4.7)	257 (8.7)
	Farmer	1,238 (21.3)	192 (6.3)	1,091 (36.8)
	Fulltime worker	348 (5.8)	165 (5.4)	183 (6.2)
	Daily worker	655 (10.9)	226 (7.4)	429 (14.5)
	Employee	688 (11.4)	555 (18.1)	133 (4.5)
	Self-employed	2,094 (34.7)	1,305 (42.6)	789 (26.6)
	Retired	565 (9.4)	479 (15.6)	86 (2.9)
**Mother's job**	Housewife	5,920 (92.8)	2,897 (89.7)	3,023 (95.9)
	Self-employed	158 (2.5)	99 (3.1)	59 (1.9)
	Employee	303 (4.7)	233 (7.1)	70 (2.2)
**Socio-economic status**	Low	2,569 (40.1)	1,011 (31.1)	1,558 (49.3)
	Middle	3,513(54.8)	1,965 (60.4)	1,548 (49.0)
	High	328 (5.1)	275 (8.5)	53 (1.7)

Qualitative variables (ordinal and nominal variables) are reported as *N* (%) and quantitative variables as mean ± SD.

### Nutritional behavior and consumption

The majority of households (65.7%) used traditional flatbreads, more than half of the households (53.9%) consumed low-fat milk and dairy products, and liquid/cooking oil was the most consumed oil within households (65.9%). Frying was the most prevalent method of cooking (54.2%). In addition, the iodized salt was used in 97.2% of households in comparison with non-iodized salt (2.8%), and storage of salt was in optimal condition in 72.0% of households for total population. Table 2 represents household nutritional behavior in total population based on the place of residence.

**Table 2:** Household Food habits in total population as well as place of residence.

		**Total (*n* = 6,429)**	**Urban areas (*n* = 3,261)**	**Rural areas (*n* = 3,168)**	***p*-value**
**Consumed bread**	Traditional flat breads	44,224 (65.7)	2,790 (85.6)	1,434 (45.3)	0.001*
	Homemade bread	2,205 (34.3)	471 (14.4)	1,734 (54.7)	
**Milk and dairy**	Low fat	3,454 (53.9)	2,066 (63.4)	1,388 (44.0)	0.001*
	High fat	2,960 (46.1)	1,191 (36.6)	1,769 (56.0)	
**Consumption of salad, lettuce and vegetable (weakly)**	Never	223 (3.5)	96 (2.9)	127 (4.0)	0.001*
	1–2 times	1,749 (27.3)	792 (24.3)	957 (30.3)	
	3–4 times	1,884 (29.4)	964 (29.6)	920 (29.1)	
	5–6 times	575 (9.0)	310 (9.5)	26 (8.4)	
	Daily	1,983 (30.9)	1,095 (33.6)	888 (28.1)	
**Oil type**	Liquid/cooking oil	4,234 (65.9)	2,273 (69.7)	1,961 (61.9)	0.001*
	Frying oil	3,818 (59.4)	2,075 (63.3)	1,743 (55.0)	0.001*
	Solid/semi-solid vegetable oil	3,241 (50.4)	1,283 (39.3)	1,958 (61.8)	0.001*
	Animal oil/animal butter	763 (11.9)	294 (0.9)	469 (14.8)	0.001*
	Olive oil	738 (11.5)	565 (17.3)	173 (5.5)	0.001*
	Canola oil	56 (0.87)	45 (1.4)	11 (0.3)	0.001*
**Methods of food preparation**	Boiled	968 (15.1)	564 (17.3)	404 (12.8)	0.001*
	Grilled	84 (1.3)	46 (1.4)	38 (1.2)	
	Steamer / oven / microwave	123 (1.9)	86 (2.6)	37 (1.2)	
	Roasted	1,762 (27.4)	994 (30.5)	768 (24.3)	
	Fried	3,477 (54.2)	1,564 (48.0)	1,913 (60.5)	
	Other	5 (0.1)	2 (0.1)	3 (0.1)	
**Iodized salt**	No	182 (2.8)	96 (2.9)	86 (2.7)	0.580
	Yes	6,247 (97.2)	3,165 (97.1)	3,082 (97.3)	
**Salt storage method**	Optimal	4,623 (72.0)	2,350 (72.2)	2,273 (71.8)	0.357
	Undesirable	1,795 (28.0)	904 (27.8)	891 (28.2)	
**Adding salt at the table**	No	4,281 (66.7)	2,194 (67.5)	2,087 (66.0)	0.207
	Yes	2,134 (33.3)	1,058 (32.5)	1,076 (34.0)	
**Add salt before tasting food**	No	5,472 (85.6)	2,763 (85.3)	2,709 (86.0)	0.464
	Yes	917 (14.4)	475 (14.7)	442 (14.0)	
**Eating out**	Yes	2,418 (37.6)	1,540 (47.2)	878 (27.7)	0.001*
	No	4,011 (62.4)	1,721 (52.8)	2,290 (72.3)	
**Fast food usage frequency**	Never	4,327 (67.5)	1,913 (58.5)	2,414 (76.4)	0.001*
	Weekly	258 (4.0)	197 (6.1)	61 (1.9)	
	Monthly	1,169 (18.2)	784 (24.1)	385 (12.2)	
	Annually	658 (10.3)	358 (11.0)	300 (9.5)	
**Use of industrial foods** **Packaged food**	Never	4,714 (73.5)	2,324 (71.5)	2,390 (75.6)	0.200
	Weekly	270 (4.2)	153 (4.7)	117 (3.7)	
	Monthly	969 (15.1)	529 (16.3)	440 (13.9)	
	Annually	460 (7.2)	246 (7.6)	214 (6.8)	

Data reported as n (%).

^*^ Significant at 0.05 level.

As shown in Table 2, there was a significant difference between rural and urban areas in terms of food habits, such as consumed bread, milk and dairy, and oil type (*p* < 0.001). Food preparation methods, eating out, and fast-food consumption were significantly different between urban and rural communities (*p* < 0.001).

### Factors associated with nutritional behavior and consumption

Higher levels of education of the head of the household and his/her spouse as well as higher SES were significantly associated with reduced consumption of home-made bread, consumption of high-fat dairy products, and frying food. In addition, the larger family size was associated with increased consumption of high-fat dairy products and frying food (*p* < 0.05). According to our results, a higher level of education was significantly associated with household iodized salt intake (odds ratio [OR] = 1.23, 95% CI: 1.08, 1.39). Moreover, a higher level of education of the head of the household and his/her spouse was significantly associated with salt storage in optimal conditions as well as not using a salt shaker at the table and not using salt before tasting food. Larger family size was associated with salt storage in undesirable conditions (OR = 0.95, 95% CI: 0.91, 0.98), using a salt shaker at the table (OR = 1.09, 95% CI: 1.05, 1.14), and using salt before tasting food (OR = 1.13, 95% CI: 1.08, 1.18). Higher SES was associated with not using a salt shaker at the table (OR = 0.89, 95% CI: 0.82, 0.98). Moreover, higher levels of education of the head of the household and his/her spouse as well as higher SES were significantly associated with increased consumption of outdoor food and fast foods. In addition, a larger family size was associated with increased consumption of fast foods (OR = 1.10, 95% CI: 1.06, 1.15). Table 3 represents the association between dietary habits and related factors.

**Table 3:** Factors associated with food habits in total population.

		**Family size**			**Mother's education level**			**Father's education level**			**Socio–economic status**		
		**OR**	**95% CI**	***p-*value**	**OR**	**95% CI**	***p-*value**	**OR**	**95% CI**	***p-*value**	**OR**	**95% CI**	***p-*value**
**Consumed bread**	Traditional flat breads	Ref.	**–**	**–**	Ref.	**–**	**–**	Ref.	**–**	**–**	Ref.	**–**	**–**
	Home–made	1.02	0.98**–**1.06	0.222	0.68	0.65**–**0.71	0.001*	0.68	0.65**–**0.71	0.001*	0.68	0.62**–**0.75	0.001*
**Milk and dairy**	Low fat	Ref.	**–**	**–**	Ref.	**–**	**–**	Ref.	**–**	**–**	Ref.	**–**	**–**
	High fat	1.13	1.09**–**1.18	0.001*	0.86	0.83**–**0.90	0.001*	0.84	0.81**–**0.87	0.001*	0.79	0.72**–**0.68	0.001*
**Methods of food preparation**	Not fried	Ref.	**–**	**–**	Ref.	**–**	**–**	Ref.	**–**	**–**	Ref.	**–**	**–**
	Fried	1.02	1.15**–**1.24	0.001*	0.90	0.87**–**0.94	0.001*	0.91	0.88**–**0.95	0.001*	0.74	0.68**–**0.80	0.001*
**Iodized salt**	No	Ref.	**–**	**–**	Ref.	**–**	**–**	Ref.	**–**	**–**	Ref.	**–**	**–**
	Yes	0.98	0.88**–**1.09	0.798	1.15	0.98**–**1.24	0.098	1.23	1.08**–**1.39	0.001*	1.07	0.83**–**1.39	0.592
**Salt storage method**	Undesirable	Ref.	**–**	**–**	Ref.	**–**	**–**	Ref.	**–**	**–**	Ref.	**–**	**–**
	Optimal	0.95	0.91**–**0.98	0.011*	1.08	1.03**–**1.13	0.001*	1.06	1.02**–**1.16	0.004*	1.04	0.94**–**1.14	0.432
**Adding salt at the table**	No	Ref.	**–**	**–**	Ref.	**–**	**–**	Ref.	**–**	**–**	Ref.	**–**	**–**
	Yes	1.09	1.05**–**1.14	0.001*	0.87	0.84**–**0.91	0.001*	0.86	0.83**–**0.91	0.001*	0.89	0.82**–**0.98	0.017*
**Add salt before tasting food**	No	Ref.	**–**	**–**	Ref.	**–**	**–**	Ref.	**–**	**–**	Ref.	**–**	**–**
	Yes	1.13	1.08**–**1.18	0.001*	0.94	0.89**–**0.99	0.034*	0.94	0.86**–**0.96	0.002*	1.07	0.95**–**1.21	0.250
**Eating out**	No	Ref.	**–**	**–**	Ref.	**–**	**–**	Ref.	**–**	**–**	Ref.	**–**	**–**
	Yes	1.03	0.99**–**1.07	0.084	1.53	1.47**–**1.60	0.001*	1.43	1.38**–**1.50	0.001*	1.93	1.76**–**2.12	0.001*
**Fast food usage**	No	Ref.	**–**	**–**	Ref.	**–**	**–**	Ref.	**–**	**–**	Ref.	**–**	**–**
	Yes	1.10	1.06**–**1.15	0.001*	1.41	1.35**–**1.48	0.001*	1.34	1.28**–**1.41	0.001*	1.72	1.55**–**1.90	0.001*

^*^ Significant at 0.05 level; CI: confidence interval.

The association between sociodemographic characteristics of households and food habits in urban and rural areas is reported in Tables 4, 5, respectively. According to the results, in rural areas, a higher level of education was associated with household iodized salt intake, but there was no association between education and iodized salt intake in urban areas. Larger family size was associated with increased consumption of home-made bread in urban areas, but there was no association between size of family and consumption of home-made bread in rural areas.

**Table 4:** Factors associated with food habits in urban population.

		**Family size**			**Mother's education level**			**Father's education level**			**Socio–economic status**		
		**OR**	**95% CI**	***p-*value**	**OR**	**95% CI**	***p-*value**	**OR**	**95% CI**	***p-*value**	**OR**	**95% CI**	***p-*value**
	OR	95% CI	*p-*value	OR	95% CI	*p-*value	OR	95% CI	*p-*value	OR	95% CI	*p-*value
**Consumed bread**	Traditional flat breads	Ref.	–	–	Ref.	–	–	Ref.	–	–	Ref.	–	–
	Home–made	0.88	0.82–0.96	0.003*	0.81	0.76–0.88	0.001*	0.83	0.77–0.89	0.001*	1.07	0.91–1.23	0.398
**Milk and dairy**	Low fat	Ref.	–	–	Ref.	–	–	Ref.	–	–	Ref.	–	–
	High fat	1.18	1.11–1.25	0.001*	0.91	0.86–0.96	0.001*	0.91	0.86–0.96	0.001*	0.88	0.78–0.99	0.048*
**Methods of food preparation**	Not fried	Ref.	–	–	Ref.	–	–	Ref.	–	–	Ref.	–	–
	Fried	1.25	1.18–1.32	0.001*	0.92	0.87–0.96	0.001*	0.94	0.89–0.99	0.030*	0.80	0.71–0.90	0.001*
**Iodized salt**	No	Ref.	–	–	Ref.	–	–	Ref.	–	–	Ref.	–	–
	Yes	1.03	0.88–1.22	0.673	1.02	0.87–1.18	0.843	1.14	0.97–1.35	0.100	0.93	0.65–1.32	0.684
**Salt storage method**	Undesirable	Ref.	–	–	Ref.	–	–	Ref.	–	–	Ref.	–	–
	Optimal	0.94	0.88–0.99	0.043*	1.07	1.01–1.14	0.014*	1.07	1.01–1.14	1.015*	1.06	0.93–1.21	0.365
**Adding salt at the table**	No	Ref.	–	–	Ref.	–	–	Ref.	–	–	Ref.	–	–
	Yes	1.15	1.08–1.21	0.001*	0.84	0.80–0.89	0.001*	0.84	0.79–0.89	0.001*	0.82	0.72–0.92	0.002*
**Add salt before tasting food**	No	Ref.	–	–	Ref.	–	–	Ref.	–	–	Ref.	–	–
	Yes	1.21	1.13–1.31	0.001*	0.91	0.85–0.98	0.020*	0.87	0.81–0.94	0.001*	1.02	0.86–1.19	0.849
**Eating out**	No	Ref.	–	–	Ref.	–	–	Ref.	–	–	Ref.	–	–
	Yes	1.01	0.95–1.04	0.759	1.46	1.38–1.54	0.001*	1.34	1.27–1.42	0.001*	1.67	1.48–1.89	0.001*
**Fast food usage**	No	Ref.	–	–	Ref.	–	–	Ref.	–	–	Ref.	–	–
	Yes	1.10	1.04–1.17	0.001*	1.31	1.23–1.39	0.001*	1.23	1.16–1.30	0.001*	1.49	1.31–1.69	0.001*

^*^Significant at 0.05 level; *CI*, confidence interval.

**Table 5:** Factors associated with food habits in rural population.

		**Family size**			**Mother's education level**			**Father's education level**			**Socio–economic status**		
		**OR**	**95% CI**	***p-*value**	**OR**	**95% CI**	***p-*value**	**OR**	**95% CI**	***p-*value**	**OR**	**95% CI**	***p-*value**
	OR	95% CI	*p*-value	OR	95% CI	*p*-value	OR	95% CI	*p*-value	OR	95% CI	*p*-value
**Consumed bread**	Traditional flat breads	Ref.	–	–	Ref.	–	–	Ref.	–	–	Ref.	–	–
	Home–made	1.01	0.95–1.05	0.916	0.83	0.77–0.88	0.001*	0.82	0.77–0.88	0.001*	0.88	0.72–1.01	0.058
**Milk and dairy**	Low fat	Ref.	–	–	Ref.	–	–	Ref.	–	–	Ref.	–	–
	High fat	1.07	1.02–1.12	0.005*	1.00	0.94–1.06	0.998	0.94	0.87–1.02	0.057	0.94	0.82–1.07	0.367
**Methods of food preparation**	Not fried	Ref.	–	–	Ref.	–	–	Ref.	–	–	Ref.	–	–
	Fried	1.14	1.09–1.20	0.001*	1.02	0.96–1.09	0.487	1.04	0.97–1.11	0.283	0.80	0.70–0.91	0.001*
**Iodized salt**	No	Ref.	–	–	Ref.	–	–	Ref.	–	–	Ref.	–	–
	Yes	0.94	0.82–1.08	0.427	1.39	1.11–1.75	0.004*	1.56	1.23–1.98	0.001*	1.35	0.89–2.05	0.150
**Salt storage method**	Undesirable	Ref.	–	–	Ref.	–	–	Ref.	–	–	Ref.	–	–
	Optimal	0.95	0.91–1.01	0.113	1.12	1.03–1.19	0.006*	1.06	0.99–1.15	0.083	1.01	0.87–1.17	0.916
**Adding salt at the table**	No	Ref.	–	–	Ref.	–	–	Ref.	–	–	Ref.	–	–
	Yes	1.05	1.01–1.11	0.034*	0.90	0.84–0.96	0.003*	0.89	0.84–0.96	0.003*	1.02	0.88–1.17	0.824
**Add salt before tasting food**	No	Ref.	–	–	Ref.	–	–	Ref.	–	–	Ref.	–	–
	Yes	1.07	1.01–1.15	0.033*	0.95	0.86–1.04	0.264	0.93	0.85–1.02	0.146	1.13	0.93–1.36	0.208
**Eating out**	No	Ref.	–	–	Ref.	–	–	Ref.	–	–	Ref.	–	–
	Yes	1.11	1.05–1.17	0.001*	1.41	1.31–1.51	0.001*	1.31	1.22–1.41	0.001*	1.76	1.51–2.04	0.001*
**Fast food usage**	No	Ref.	–	–	Ref.	–	–	Ref.	–	–	Ref.	–	–
	Yes	1.20	1.13–1.28	0.001*	1.26	1.15–1.37	0.001*	1.19	1.08–1.31	0.001*	1.44	1.19–1.73	0.001*

^*^Significant at 0.05 level; *CI* confidence interval.

## Discussion

In this study, liquid/cooking oils were the most consumed oil type among households (65.9%). In addition, olive and canola oils were consumed by 11.5 and 0.87% of households, respectively. However, consumption of liquid and vegetable oils in Sanandaj Province, Iran, was 26.75% (8). These vegetable oils have shown beneficial properties toward cardiovascular diseases due to their fatty acid composition. Consuming more than 2.5 times mono-unsaturated fatty acids (MUFAs) and poly-unsaturated fatty acids (PUFAs) present in vegetable oils (9) than saturated fatty acids (SFAs) is considered a healthy behavior in the Healthy Eating Index 2015 (10).

In contrast to liquid and vegetable oils, animal meat and high-fat dairy products contain high amounts of SFA. SFA consumption is responsible for various chronic conditions, including cardio heart disease (CHD) (11); thus, it is recommended by the Dietary Guideline for Americans (DGA) to use <10% of daily calorie intake from SFA and replace it with PUFA (11).

Dietary guidelines suggest reducing the consumption of high-fat dairy products (12). In the present survey, almost nearly half of the households (53.9%) consumed low-fat milk and dairy. Although low-fat milk and dairy products were significantly higher, it was observed that a significant difference could be due to the large sample size. As the American meta-analysis reported, high-quality evidence supports favorable associations (i.e., decreased risk) between total dairy intake and hypertension risk and between low-fat dairy and yogurt intake and reduced risk of type 2 diabetes (T2D) and the consequent massive burden of health economics in this area (13). Thus, proper methods of awareness are required to amend dairy product consumption patterns in this area.

In this study, the most common methods of cooking were fried cooking (54.2%). Deep frying with oil negatively changes the fatty acid composition of oil. Frying increases energy density and decreases the water content of the meal. In a case-control study in India, patients with coronary heart disease, when compared with the control group, reported a greater intake of shallow fried food. Data from a case-control study in China showed that the frequency of fried food intake was significantly higher in patients with acute myocardial infarction. Data from the Nurses' Health Study and the Health Professional Follow-Up Study showed that frequent fried food consumption was significantly associated with a higher risk of coronary artery disease (CAD) (14). Thus, health and nutrition education as well as the improvement of healthy snacks and physical activity, especially at schools and kindergarten can change nutritional behaviors in Iranian households.

Iodine plays an essential role in the functions of thyroid hormones. The prevention of iodine deficiency disorders (IDDs) is one of the most important health programs in Iran. Based on our results, 97.2% of households used iodized salt. The World Health Organization (WHO) and International Council for Iodine Deficiency Disorders (ICCIDD) standards state that the elimination of IDD will be possible if more than 90% of households consume adequately iodized salt (15). The Iodized Salt Coverage Study 2010 shows that the availability of adequately iodized salt in the households in Orissa has almost doubled from 32.4%, conducted by the National Food and Health Survey (NFHS) 3 in 2005–2006 to 59% in 2010. Consumption of iodized salt has increased, but it is still way behind the universal salt iodization (USI) target of 90% of households consuming adequately iodized salt (16, 17). In a study by Srivastava et al., two-thirds (65.2%) of the households were adequately consuming iodized salt, while about one-fifth (21.5%) of the households were consuming iodized salt inadequately (18).

### The association between nutritional behavior and level of education

In this study, a higher level of education was significantly associated with reduced consumption of fried food and high-fat dairy products, although, in such families, traditional flatbreads and fast food were consumed more.

In addition, a large-scale meta-analysis investigated 15 European countries and determined that the parents' higher level of education was associated with healthy diet behaviors. A similar result was reported in a Danish survey too (19). The analysis of demographic and socioeconomic data determined that education is usually the strongest component of socioeconomic differences (20). In contrast, Aslam et al. reported that consumption of all groups, healthy and unhealthy foods such as sugar and fast foods, was highly associated with the level of education (21).

In this study, a higher level of education of the head of the household and his/her spouse was significantly associated with iodized salt storage in optimal conditions as well as not using a salt shaker and not using salt before tasting food. Valexi et al. showed that women and men with a high level of education knew how to store iodized salt (22). However, Azizi et al. showed that the level of education was not significantly associated with the storage of salt (23). Higher levels of education may also improve the ability to believe or understand health-related information, in general, or dietary practice, in particular, needed to develop health-promoting skills and beliefs in the field of food habits. It is believed that with a higher level of education, there is more knowledge about healthy food items (20, 24).

## The association between nutritional behavior with socioeconomic status and family size

In this study, higher SES was significantly associated with reduced consumption of fried food and high-fat dairy products but fast food/outdoor food was increased. A similar result was found in the Kelishadi's study, suggesting that families with higher SES had healthier dietary practices (2). Esghinia et al. (23)and Rezazadeh et al. (25) found a rising trend toward healthy and nutritive behaviors with the increase in SES.

According to previous studies, SES is one of the most important determinants of diet quality in children and adolescents. This can be due to higher nutritional knowledge in high SES regions and limited access and affordability for some fast foods in low SES regions (26).

In this study, SES was not significantly associated with iodized salt intake. However, a study in Tehran showed that the SES influenced iodized salt consumption (27). In studies conducted by Kouhi et al. (28), Sharifirad et al. (29), and Yarmohammadi et al. (30) on students, there was a direct relationship between economic status and fast-food consumption (31). It can be concluded that good income acts as a double-edged sword. Although a favorable economic status can cause the consumption of healthy snacks and healthy nutrition, it can increase their purchasing power for junk foods.

Based on our findings, an unhealthier dietary pattern may exist among the households who are in lower socioeconomic level in Iran. The relationship between SES and nutritional performance has been studied (32). Many studies have determined that eating patterns of people in low SES groups threaten public health (33–37). People in the low SES group, due to a lack of accessibility to healthcare, improper living conditions, less education, and greater psychological stress, may be at a greater risk of poorer health status than others (38). Thus, appropriate policies, interventions, and efforts aimed at improving nutrition-related health, especially in high-risk groups, are necessary.

In this study, daily consumption of vegetables was close to 30%. A healthier dietary pattern is believed to include a higher consumption of fruits and vegetables and a lower consumption of fat and meat. Thus, people with a higher socioeconomic level tend to show a higher consumption of vegetables, fruits, and fiber products, and a lower consumption of meat, meat products, and fats in comparison with people with a lower socioeconomic level (20). Abdollahi et al. showed that with the increase in knowledge and occupational level of the heads of households, consumption of high-dense calories and lower healthy groups decreased. A large-scale meta-analysis from 15 European countries also showed that higher levels of literacy were associated with higher consumption of vegetables and fruits (1, 20, 39). One of the main principles of healthy diet is the daily intake of vegetables that are effective in preventing NCDs. Therefore, certain interventions are necessary to increase vegetable consumption per capita, including increasing access to this group of food and promoting vegetable consumption from childhood.

In this study, family size was associated with increasing consumption of fast food, high-fat dairy products, and fried food, while larger family size was significantly associated with iodized salt storage in undesirable condition as well as using a salt shaker and using salt before tasting food. It is obvious that family size can affect dietary behaviors in the household. When family size increases, the eating habits of a person in the family can affect all members of the family and show itself as the eating behavior of the family. It is to be expected that family members have different eating habits, but unhealthy eating habits are usually more pronounced than other eating habits.

### The association between food habits and place of residence

In this study, almost all eating practices were significantly different between rural and urban areas. According to the results, in rural areas, higher levels of knowledge of the head of the household and his/her spouse were associated with household iodized salt intake, but in urban areas, there was no association between knowledge of the head of the household and his/her spouse and iodized salt intake. A larger family size was associated with decreased consumption of home-made bread in urban areas, but there was no association between size of family and consumption of home-made bread in rural areas. Consumption of fast food was significant in both urban and rural areas in this study.

Kelishadi et al. did not report any significant differences between urban and rural areas for salt intake. This finding is probably because of the main sources of salt intake, i.e., bread, cheese, and many junk foods such as cheese puffs and potato chips, which are regularly consumed by all Iranian people and are not limited to urban areas or a kind of socioeconomic categories. On the contrary, a survey in Ethiopia and Sudan showed that individuals in urban areas were 9 times more likely to be aware of iodized salt consumption than those who lived in rural areas (40).

Participants in regions with higher SES had healthier nutritional behaviors, but some nutritional behaviors, such as consumption of fast food less often, were similar in areas with the lowest and highest SES. This can be due to higher health knowledge in high SES regions, and inaccessibility and limited affordability for some fast foods in low SES regions (2).

Despite the expectation that increased knowledge of the head of the household and his/her spouse may create a negative attitude toward eating unhealthy food by being more aware of the importance of healthy nutrition for families, the tendency toward consuming more unhealthy diets and fast food, especially among the adolescents, has increased in households in today's urban communities as a result of longer working hours, more busy educated parents in outdoor work environments, as well as time limitations.

### Study strengths and limitations

One of the strengths of this study is that it was a comprehensive study with a large sample size, so that the samples represent the whole community. In addition, this study examined all aspects of household dietary behaviors and considered all factors affecting eating habits. The main limitation of this study was that we did not assess the disease and anthropometric data of the households. Therefore, future studies are suggested to obtain more information, including disease and anthropometric data.

## Conclusion

Most households follow healthy practices, especially types of oils consumed, methods of preparing their food as well as keeping salt in an optimal condition and consuming iodized salt. The most important unhealthy nutritional behavior was the high consumption of fast food and outdoor food, especially in urban regions. Unhealthy nutritional behaviors were more prevalent in households with low household and regional SES. It is suggested to consider community nutrition education and socioeconomic disparities should be considered for public health interventions aiming to improve food habits.

## Data availability statement

The original contributions presented in the study are included in the article/supplementary material, further inquiries can be directed to the corresponding author.

## Ethics statement

This study was conducted according to the guidelines laid down in the Declaration of Helsinki and all procedures involving research study participants were approved by the Shiraz University of Medical Sciences (SUMS), Fars, Iran, under the registration code: IR.SUMS.REC.13940598. The consent form was pre-designed and for all interviewed households, before the research questions, the consent form was completed and if the head of the household or the interviewee was satisfied, the study questionnaire was completed. The patients/participants provided their written informed consent to participate in this study.

## Author contributions

ZF: analysis and interpretation of data and writing the original draft. MM: writing the original draft. MAM: research idea and study design. AH: questionnaire design, analysis interpretation of data, and review and editing. AF: collect and clear data and review editing. MA: research idea and study design. RS: research idea and study design, patient enrolment, and writing the original draft. All authors have read the final version of the manuscript and approved it.

## Funding

This project was discussed by the research committee of the Shiraz University of Medical Sciences and approved, and the proposed budget was allocated in the proposal through the same committee in several stages and after the submission of the work report.

## Conflict of interest

The authors declare that the research was conducted in the absence of any commercial or financial relationships that could be construed as a potential conflict of interest.

## Publisher's note

All claims expressed in this article are solely those of the authors and do not necessarily represent those of their affiliated organizations, or those of the publisher, the editors and the reviewers. Any product that may be evaluated in this article, or claim that may be made by its manufacturer, is not guaranteed or endorsed by the publisher.
